# Validity of the Maximal Heart Rate Prediction Models among Runners and Cyclists

**DOI:** 10.3390/jcm12082884

**Published:** 2023-04-14

**Authors:** Przemysław Seweryn Kasiak, Szczepan Wiecha, Igor Cieśliński, Tim Takken, Jacek Lach, Marcin Lewandowski, Marcin Barylski, Artur Mamcarz, Daniel Śliż

**Affiliations:** 1 3rd Department of Internal Medicine and Cardiology, Medical University of Warsaw, 04-749 Warsaw, Poland; 2 Department of Physical Education and Health, Faculty in Biala Podlaska, Jozef Pilsudski University of Physical Education in Warsaw, 21-500 Biala Podlaska, Poland; 3 Department of Medical Physiology, Child Development & Exercise Center, Wilhelmina Children’s Hospital, UMC Utrecht, 3584 EA Utrecht, The Netherlands; 4 Department of Pharmacology and Clinical Pharmacology Collegium Medicum, Cardinal Stefan Wyszyński University in Warsaw, 01-938 Warsaw, Poland; 5 Department of Internal Medicine and Cardiac Rehabilitation, Medical University of Lodz, 90-549 Łódź, Poland

**Keywords:** maximal heart rate, endurance athletes, cardiopulmonary exercise test, exercise physiology, prediction models, endurance performance

## Abstract

Maximal heart rate (HRmax) is a widely used measure of cardiorespiratory fitness. Prediction of HRmax is an alternative to cardiopulmonary exercise testing (CPET), but its accuracy among endurance athletes (EA) requires evaluation. This study aimed to externally validate HRmax prediction models in the EA independently for running and cycling CPET. A total of 4043 runners (age = 33.6 (8.1) years; 83.5% males; BMI = 23.7 (2.5) kg·m^−2^) and 1026 cyclists (age = 36.9 (9.0) years; 89.7% males; BMI = 24.0 (2.7) kg·m^−2^) underwent maximum CPET. Student *t*-test, mean absolute percentage error (MAPE), and root mean square error (RMSE) were applied to validate eight running and five cycling HRmax equations externally. HRmax was 184.6 (9.8) beats·min^−1^ and 182.7 (10.3) beats·min^−1^, respectively, for running and cycling, *p* = 0.001. Measured and predicted HRmax differed significantly (*p* = 0.001) for 9 of 13 (69.2%) models. HRmax was overestimated by eight (61.5%) and underestimated by five (38.5%) formulae. Overestimated HRmax amounted to 4.9 beats·min^−1^ and underestimated HRmax was in the range up to 4.9 beats·min^−1^. RMSE was 9.1–10.5. MAPE ranged to 4.7%. Prediction models allow for limited precision of HRmax estimation and present inaccuracies. HRmax was more often underestimated than overestimated. Predicted HRmax can be implemented for EA as a supplemental method, but CPET is the preferable method.

## 1. Introduction

Maximal heart rate (HRmax) is a widely used variable to recommend training intensity by exercise practitioners [1] and prescribe treatment by medical professionals [2]. HRmax usually means the highest heart rate (HR) achieved during maximum symptom-limited cardiopulmonary exercise test (CPET) [3]. Verification of achieving HRmax can be carried out based on volitional CPET termination or by HR plateau (lack of growth in the HR with increasing intensity) [4,5]. Currently performed CPET often leads to non-diagnostic results. Participants terminate them at the submaximal intensity (without signs of ischemia or <85% of HRmax) based only on prespecified HRmax. The clinical value of such CPET is sub-optimal, which was confirmed by ECG and imaging data [4,5].

HRmax shows significant variability [1,6]. Measuring the HR during exercises and calculating its proportion to HRmax, resting HR, or relative workload (% of HRmax) is a common practice. It has been implemented in many wearable devices [6]. Results help to optimize exercise intensity for both healthy endurance athletes (EA) and rehabilitating patients. It has been confirmed that people with better fitness levels, in particular EA, can achieve higher HRmax and maintain slower declines in HRmax with aging [6,7]. They also have lower all-cause mortality, especially due to CVD [6].

CPET results differ significantly between treadmill and cycle modality [8]. The differences apply not only to HRmax but also to other performance indicators [9,10]. Usually, higher scores are observed in running CPET [9]. Cycling CPET has a lower occurrence of attributes because it is performed in a more stable position [8]. In consequence, the cycling test is recommended more often when higher measurement accuracy is required (e.g., in the clinical settings) [3].

There is a demand for individualized, risk-based, stratified therapy and exercise programs. This points out that medical decisions, intensity prescriptions, and diagnostic examinations are precisely tailored to the individual [11,12]. Knowledge of the endurance capacity therefore remains essential. Prediction models are usually developed to share decision-making [11]. Numerous regression equations have been derived to obtain the HRmax setpoint without the maximal CPET [13]. However, their accuracy is often questioned and there is a lack of large studies comparing different prediction models among EA and stratifying them between CPET modalities. Recently, endurance sports have been gaining popularity, hence the number of EAs with suspected CVD and veteran EAs increased. Knowledge of the exact value of HRmax is particularly important for them.

The majority of prediction models include a baseline value (around 200–220) and different age covariates [14]. Their authors postulate that such a univariate analysis with the inclusion of only one key factor is sufficient and other variables remain negligible [2]. Examples of the most commonly used models are those provided by Fox et al. [15] (220 age) and Tanaka et al. [2] (207.5–0.7 age). They are well known and highly popular. Many physicians and fitness practitioners use them for the evaluation of exercise intensities. Their advantage is the simplicity of calculations that can be performed by anyone in the field, clinical or home settings. However, they have some disadvantages. This may lead to inaccurate HRmax predictions and is not fully transferable [14]. Other authors are looking for a more optimal ratio of variables adjusted for other populations or testing modalities [16,17,18]. Due to the emergence of new HRmax prediction models, their accuracy and transferability remain unvalidated and need verification.

Indirect estimation of HRmax has found wide application in fitness equipment, and it is a common method for preparing medical recommendations (e.g., for treatment of heart failure) [1,19]. The use of inaccurate HRmax predictions could lead to providing unreliable exercise recommendations and ineffective training plans for athletes [20]. For patients, incorrectly estimated HRmax values may result in suboptimal rehabilitation, inappropriate risk stratification, and pose a health risk [21,22]. Moreover, there is a lack of studies on large cohorts that verify the general precision of the given models and confirm their universal usage.

In summary, this study seeks to (1) assess the overall accuracy of HRmax prediction models in the general population of healthy EA independently for running and cycling CPET, (2) evaluate their practical application based on the precision of their estimations, and (3) to provide further research recommendations to improve the accuracy of novel prediction models. By conducting the first external validation of HRmax prediction models stratified between CPET types on a large population of endurance athletes, this study aims to contribute valuable insights to the field, which may inform the development of more accurate HRmax prediction models and support better decision-making in both athletic and clinical settings.

## 2. Materials and Methods

### 2.1. Study Setting

**Figure 1:**
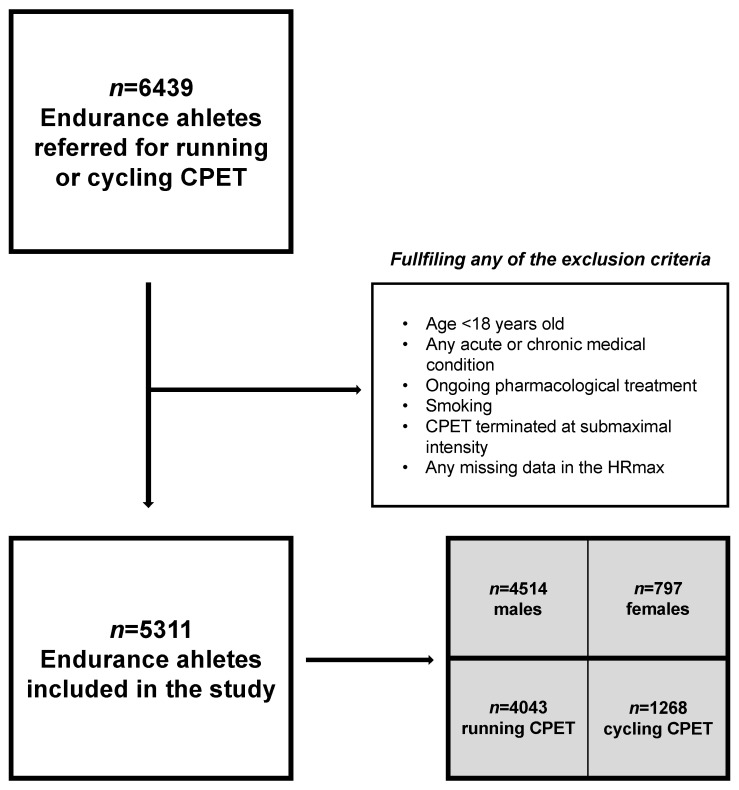
Flow diagram for inclusion procedure. Abbreviations: CPET, cardiopulmonary exercise test; HRmax, maximal heart rate. From 6439 endurance athletes screened for inclusion in the years 2013–2021, 5311 (82.48%) met the study criteria.

This is a population study on a cohort of healthy adult EA who declared regular training and had ≥3 months of endurance training experience. A retrospective analysis of CPET data collected between 2013 and 2021 from the tertiary care sports medicine center SportsLab (www.sportslab.pl, accessed on 17 March 2023; Warsaw, Poland) was performed. All CPETs were conducted at the individual request of athletes as part of the optimization of a training program or periodic performance evaluation. Exclusion criteria were: (1) age < 18 years old, (2) any medical contraindications (mild/severe and acute/chronic), (3) usage of any medications at the date of study (acutely/chronically), (4) smoking, (5) missing data in HRmax. The selection procedure is presented in Figure 1. Validation was performed following the TRIPOD guidelines (see Supplementary Materials File S1: TRIPOD Checklist: Prediction Model Development and Validation) [23].

### 2.2. Previously Published HRmax Prediction Models

Candidates of prediction models were identified from systematic reviews for normative exercise reference values by Paap et al. and Takken et al. up to 2019 [24,25]. Screening for models derived between 2019 and 2023 was conducted by a manual literature search in four electronic databases, PubMed, MEDLINE, Scopus, and Web of Science, using keywords “prediction model”, “prediction equation”, “prediction algorithm”, “endurance athletes”, “cardiopulmonary exercise testing” and “maximal heart rate”. Inclusion criteria were: (1) usage only of somatic or exercise variables which were available in our database, (2) providing HRmax defined as the peak value (not averaged), and (3) providing data about the primarily derived population stratified by CPET modality. Exclusion criteria were: (1) being derived primarily for pediatric or geriatric populations, (2) being derived exclusively for one sex, and (3) focusing on clinical population. Additionally, Fox et al. [15] and Tanaka et al. [2] equations were added due to their wide usage. Finally, 13 different prediction models from 9 studies were qualified. Their classification is presented along with their accuracy results. Original derivation studies are presented in Supplementary Materials File S3: Selected prediction models for maximal heart rate.

### 2.3. Cardiopulmonary Exercise Testing Procedures

All CPETs were performed in a single laboratory under unified protocols both for treadmill and cycle ergometry. EA underwent graded maximal effort CPET on either a mechanical treadmill (h/p/Cosmos quasar, Nussdorf–Traunstein, Germany) or cycle ergometer (Cyclus 2, RBM elektronik-automation GmbH, Leipzig, Germany). Briefly, the testing modality was selected by the agreement of the subject and the physiologist to suit the primary training discipline.

The cycling CPET started with a 5 min free wheel pedaling as a warmup and continued with a gradual increase in resistance every 2 min until termination (20 W for females and 30 W for males). The running CPET began with a 5 min walking or slow jogging warmup and continued with a gradual increase in velocity every 2 min (1 km·h^−1^ for both females and males). The treadmill was set at a constant inclination equal to 1%. The intensity was adjusted by the physiologist in conjunction with the trainee to reach their maximum exertion. The termination points considered indicative of maximal effort included (1) volitional exhaustion and inability to continue the protocol with declared exertion ≥ 18 in Borg’s RPE, and (2) an HR or oxygen uptake (VO_2_) plateau (a stable level of HR or leveling-off in VO_2_, defined as an increase < 100 mL·min^−1^ with growing exercise intensity before CPET termination) [26,27,28].

Exercise indices were obtained breath-by-breath by the Hans Rudolph V2 Mask (Hans Rudolph, Inc., Shawnee, KS, USA), a gas exchange monitor Cosmed Quark CPET (Rome, Italy), and analyzed using dedicated software Omnia. HR was measured via ANT+ chest strap as a part of the Cosmed Quark CPET set (manufacturer product accuracy comparable to ECG; ±1 beats·min^−1^). HRmax was defined as the peak value and was not averaged in the interval preceding CPET termination. Maximal VO_2_ (VO_2_max) was considered as the average VO_2_ during the 15 s period at the end of the CPET. The maximal oxygen pulse was calculated as VO_2_max/HRmax, maximal respiratory exchange ratio was calculated as the maximal volume of exhaled carbon dioxide/maximal volume of oxygen uptake, and maximal minute ventilation efficiency was calculated as maximal ventilation/maximal volume of exhaled carbon dioxide.

### 2.4. Data Analysis

Continuous variables are presented as mean (standard deviation; SD). In total, 95% confidence intervals (CI) were calculated for predicted HRmax and the difference between observed and predicted values. Categorical variables are presented as numbers (percentages). Equations were tested independently for running and cycling CPET. Data distribution was assessed by the quantile–quantile plots. Differences between both disciplines (running/cycling) were calculated by Student *t*-test. All numerical data are presented following the American Medical Association (AMA) guidelines.

The predictive of the selected formulae were compared by the MAPE (mean absolute percentage error) and RMSE (root mean square error). MAPE indicates function loss by a regression model. RMSE presents the unbiased value of prediction errors because it is consistent during the assessment of given models. Both measures are intuitive to interpret the relative inaccuracy of prediction. RMSE was additionally adjusted to the percentage of observed HRmax (by dividing the error by the mean of observed HRmax). Differences between observed and predicted HRmax for each prediction model were also calculated by Student *t*-test.

The significance agreement was adopted at two-tailed *p* = 0.05. Statistical analyzes were performed in the IBM SPSS Statistical Software (version 29.0, IBM, Chicago, IL, USA).

## 3. Results

### 3.1. Athletes’ Characteristics

The cohort consisted of 5311 EA. The cohort included 4043 running CPETs (3377 males, 83.53%) and 1268 cycling CPETs (1137 males, 89.67%). The age of the runners was 33.58 (8.12) years and the age of the cyclists was 36.88 (9.03) years, *p* < 0.001. Participants were classified as having normal weight; the BMI of runners was 23.66 (2.54) kg·m^−2^ and that of cyclists was 24.04 (2.65) kg·m^−2^, *p* < 0.001.

During the CPET, HRmax was 184.60 (9.79) beats·min^−1^ and 182.66 (10.28) beats·min^−1^, respectively, for runners and cyclists, *p* < 0.001. VO_2_max also differed significantly between modalities (53.24 (7.12) mL·min^−1^·kg^−1^ for runners; 51.67 (7.86) mL·min^−1^·kg^−1^ for cyclists), *p* < 0.001. All remaining variables, except body fat percentage (*p* = 0.09) and maximal oxygen pulse (*p* = 0.53), differed significantly between test types, all *p* < 0.001. Full demographic and CPET results stratified by sex and testing modality are summarized in Table 1.

### 3.2. Performance of Selected Prediction Equations HRmax

Among the selected equations, 66% (five of eight for treadmill and three of five for cycle ergometer) underestimated HRmax in our athletic cohort and values ranged from 0.18 beats·min^−1^ (CI = 184.17, 184.67; MAPE = 4.35%) for Fox et al. in running CPET up to 4.90 beats·min^−1^ (CI = 177.46, 178.08; MAPE = 4.68%) for Fairbarn et al. in cycling CPET. Among the selected equations, 33% (three of eight for treadmill and two of five for cycle ergometer) overestimated HRmax and values ranged from 0.08 beats·min^−1^ (CI = 182.39, 183.11; MAPE = 4.10%) for Arena et al. in cycling CPET up to 4.94 beats·min^−1^ (CI = 189.34, 189.74; MAPE = 4.94%) for Machado et al. in running CPET. Significant differences with *p* < 0.001 were observed less frequently in equations derived for treadmill CPET (seven of eight) compared to cycling CPET (two of five).

The lowest accuracy has been noted for the Machado et al. running formula (RMSE = 10.47, %RMSE = 5.67) and cycling equation provided by Fairbarn et al. (RMSE = 10.38, %RMSE = 5.68). Selected models explained variability in HRmax equal to 9.84 beats·min^−1^ when considering an absolute range of positive and negative values. Student *t*-test indicated that the predicted HRmax differed significantly among all equations compared to the observed HRmax (all *p* < 0.001), except Fox et al. (*p* = 0.38 and *p* = 0.23 for running and cycling CPET, respectively), Tanaka et al. (*p* = 0.16 for cycling CPET) and Arena et al. (*p* = 0.80 for cycling CPET). MAPE values ranged between 3.95 and 4.69%. A complete analysis of the prediction performance is presented in Table 2 (upper Part A and lower Part B, respectively, for running and cycling formulae). Bland–Altman plots for visual comparison of observed and predicted data are included in Supplementary Materials File S2: Prediction performance of selected maximal heart rate prediction models.

## 4. Discussion

In this retrospective analysis of data from CPET conducted at the tertiary care sports diagnostic center, we examine the relationship between directly measured and predicted HRmax by the 13 commonly used regression equations. We demonstrate that (1) overall predicted values differed significantly for the majority of formulae among general and healthy athletic cohort, (2) the underestimation between the predicted and observed values ranged from 0.18 beats·min^−1^ to 4.90 beats·min^−1^, (3) overestimation of HRmax was observed less often than underestimation among EA, and (4) overestimation ranged from 0.08 up to 4.94 beats·min^−1^. By definition, external validation is “assessing the predictive agreement of a prediction model in a research population other than the one from which the model was developed” [32]. We underline the lack of external validation studies on EA performed comprehensively for numerous HRmax equations. The main novelties of the present research are a wide cohort of EA at different levels of fitness and independent analysis adjusted for treadmill and cycling CPET. Such an approach enables reliable validation showing whether current models are transferable and suitable for EAs at both testing modalities.

### 4.1. Importance of Evaluation of HRmax Predictions

Accurate prediction of HR might be helpful to confirm that the maximal effort has been achieved, compare training intensity with the maximal capacity of the individual, or consider clinically focused CPET as valuable in diagnosing CVDs [6]. The most accurate way to obtain HRmax is to perform laboratory CPET or maximum effort during the competition [19]. Although those methods are not always accessible due to the limited availability of specialized diagnostic centers, high costs of fees, participant health restrictions, and limitations in locations of events [33]. Despite significant inaccuracy, indirect measurement is widely applied in practice. So far, the accuracy of Fox et al. [15] and Tanaka et al. [2] models have been most frequently evaluated. Along with their popularity, they already have some inaccuracies. Nes et al. postulate bias at the level of 4–7 beats·min^−1^ for Tanaka et al. and up to 35 beats·min^−1^ for Fox et al. in certain subjects [14]. Furthermore, Magri et al. report that in a clinical population consisting of patients with heart failure, Fox et al. led to 37.60% and Tanaka et al. led to 42.60% errors compared to directly measured HRmax [34]. However, a comprehensive evaluation of other previously derived models is missing, and most studies focus only on a few particular formulae.

### 4.2. Comparison and Derivation Background of Current HRmax Prediction Models

Our validation approach directed at the EA enables a comprehensive assessment of whether models are fairly replicable. Current HRmax formulae were originally derived from varied samples. Briefly, our results indicate that they do not perform precisely in EA, despite the fact that we only selected models derived from healthy, active cohorts with demographic comparable to that of our subjects. This is especially important due to their wide usage in sports diagnostics. The bias for most of the formulae ranged from 0 to 5 beats·min^−1^. However, the one provided by Machado et al. [29] overestimated HRmax for up to 4.94 beats·min^−1^. MAPE is our additional approach for the evaluation of HRmax prediction performance. MAPE indicated that Arena et al. and Tanaka et al. perform with substantial accuracy for each modality. Their MAPE was the lowest both for treadmill and cycling data. We also noticed that Arena et al. (*p* = 0.80 for cycling), Tanaka et al. (*p* = 0.16 for cycle) and Fox et al. (*p* = 0.30 for treadmill and *p* = 0.23 for cycling) equations were the only ones that did not differ significantly from measured values. Our results indicate that these models perform quite precisely for EAs. They were characterized by the lowest inaccuracies, perhaps because they were derived from numerous populations from synthesized various studies [2,15] or cohorts with above-average physical activity levels [16]. Thus, the ratio between basic (~200–220) and age covariates requires further studies to determined the most precise values.

It is worth to underline that MAPE is more biased toward clinical than healthy, athletic populations. Our MAPE was lower for the athletic cohort than for the patients with heart failure in Magri et al. [22] study (4.25% and 4.41% vs. 37.60% for Fox et al. or 3.96% and 4.11% vs. 42.60% for Tanaka et al.). This indicates that HRmax prediction could be more accurate for active compared to diseased individuals and perhaps find its wider practical application among EA. We stipulate that these results emerge other confounding variables in participants with heart failure or CVDs (i.e., impact of medications, heart anatomy, etc.) [22].

### 4.3. Consequences of Using Inaccurate HRmax

Relying on inaccurate results when setting the exercise intensity may lead to suboptimal, non-diagnostic effort [3]. As a rule, the intensity for exercise medical programs should be 70–80% of HRmax for moderate-intensity steady-state activity and >85% of HRmax for high-intensity interval training [21]. An underestimated HRmax of ~5 beats·min^−1^ (i.e., ~3% of HRmax for 30-year male individuals according to the widely used Fox et al. algorithm) does not seem to be a wide inaccuracy. This level of bias fills in the range of adjusted intensity for medical programs. Thus, predicted HRmax could be applied to EA in a medical setting, but we underline that this relationship for the general and clinical populations needs to be confirmed. It is worth providing a few specific examples of ways in which such inaccuracies might impact the effectiveness of training programs, risk of injury, or overall performance outcomes [13,20]. The incorrectly adjusted intensity of the training plan leads to the lack of observed intended benefits. Excessively low training intensity caused by the use of underestimated HRmax could prevent EA from making progress. On the other hand, excessively high intensity resulting from applying overestimated HRmax leads to overtraining and elevates the risk of injury, including both musculoskeletal and cardiovascular conditions.

So far, previous studies have usually postulated that models may underestimate HRmax in people with a higher level of fitness [14,34]. Physical activity allows maintaining high and stable HRmax, despite increasing age. In addition, people with lower endurance capacity experience a steeper decline [35]. Our results showed a similar relationship and only ~40% (i.e., three of eight for running CPET and two of five for cycling CPET) overestimated HRmax in the athletic cohort.

### 4.4. Other Possible Predictors of HRmax

All selected formulas included just two variables, i.e., age and a basic multiplier of about 200–220 with varied proportions of them. The use of age as the main somatic variable results from its dominant influence on the decrease in HRmax [2]. However, other parameters such as BMI, body composition, and body fat or VO_2_max can also affect HRmax due to differences in the ratio between fat mass and muscle mass [36,37]. It is well documented that EAs achieve higher HRmax and a slower decline in HRmax with age than the general population [7]. However, the majority of them differ significantly compared to observed values. We suggest that predicting HRmax only in this way may not be the most optimal method, and other estimation possibilities could exist. Previous reports indicate that there could be an impact of resting HR or submaximal HR at first and second ventilatory thresholds, BMI, body mass, VO_2_max, body fat, and testing modality [38,39]. All of them are parameters regularly measured by most diagnostic centers. Recently derived more specified models consider blood counts, left ventricular ejection fraction (LVEF), and diet [22]. Potential methods of their implementation may require laboratory tests for blood counts, radiologic examination for LVEF, and questionnaire nutritional assessment. However, due to practical reasons, it may be demanding to implement them in real-life circumstances. Thus, measuring those variables for all subjects could be problematic. Staying with predicting HRmax based on additional somatic and exercise variables other than the age-only approach seems to be simultaneously a more accurate and feasible tool.

### 4.5. Limitations

The study has some limitations that should be acknowledged. One notable limitation is the underrepresentation of female endurance athletes in the sample, which comprised only 15.01% of the participants. Additionally, our cohort was skewed toward an older age group. To address these limitations, we recommend that future research should aim for a more balanced representation of male and female participants, as well as include a broader age range with a higher number of younger participants. Furthermore, it would be beneficial for future studies to investigate the validity of HRmax prediction models that are specifically derived for and validated in single-sex populations to better understand any potential sex-specific differences in the accuracy of these models. It is worth emphasizing that despite the achieved statistical significance, the values of ~4% and 8 beats/min^−1^ at peak exercise are not necessarily clinically significant. Statistical significance may have occurred due to the significant sample size. Another limitation of this study is the lack of racial diversity because the population consisted only of Caucasian participants [40,41]. Thus, the generalizability of the results should be considered carefully.

### 4.6. Perspective and Further Study Directions

The accuracy of the predicted HRmax leaves considerable room for improvement. Recent reports suggest the involvement of other contributing variables, but their precise impact remains understudied. We suggest that the way to accurately predict HRmax is not by looking for the perfect ratio age-basic multiplier ratio. Perhaps including new, more advanced predictors (resting or submaximal exercise performance, past medical history, laboratory blood results, hemoglobin concentration, heart anatomy, LVEF, daily habits- diet, pharmaceutics, usage of β-blockers, etc.) may allow for more adjusted analyses [22,38,39,42,43,44]. We also recommend exploring for which samples the predictive models were least accurate and identifying potential predictors of this inaccuracy. We affirm that there may be some contribution seen from a level of fitness, training experience, or primary sport discipline. Additional analysis on narrow, more specified athletic cohorts could provide valuable insights into the practical applications of the predictive models. We recommend more detailed research to assess their relationship with HRmax and their possible inclusion in predictive modeling directly for EA.

Findings also have implications beyond the validated population. The analysis of only healthy participants with the exclusion of any possible disturbing factors allowed us to obtain clear and absolute results of HRmax predictions. This can be helpful as a reference value when comparing with individuals suffering from clinical conditions (e.g., to grade the severity of CVDs and health impairment). Moreover, such athletic reference values provide valuable insights for fitness practitioners when working with newly engaged endurance athletes whose characteristics are similar to those of the general population (e.g., to set a training goal). However, when applied in other circumstances, findings should be extrapolated and interpreted carefully.

## 5. Conclusions

We conducted external validation of 13 commonly used prediction equations for HRmax in the EA cohort. Predicted HRmax was significantly different from that observed in CPET across most models (11 of 13). Prediction models allow for limited precision of HRmax estimation and present inaccuracies. Underestimation of HRmax occurred more often than overestimation. HRmax predictions can be implemented as a supplemental method in sports diagnostics when direct measurement is not possible and cannot replace full CPET. However, medical professionals and fitness practitioners should acknowledge the remaining inaccuracies, and predicted HRmax should not be the primary, preferable way of evaluating and adjusting exercise intensity.

## Figures and Tables

**Table 1:** Participant characteristics.

Variable		Running CPET		Cycling CPET			*p*-Value
	All (n = 4043)	Males (n = 3377)	Females (n = 666)	All (n = 1268)	Males (n = 1137)	Females (n = 131)	
Age (years)	33.58 (8.12)	33.96 (8.15)	33.66 (7.67)	36.88 (9.03)	37.30 (9.11)	33.19 (7.37)	**<0.001**
Weight (kg)	74.87 (11.18)	77.68 (9.38)	60.60 (8.36)	76.99 (10.76)	78.81 (9.53)	61.18 (7.30)	**<0.001**
Height (cm)	177.52 (7.84)	179.55 (6.24)	167.22 (7.00)	178.77 (7.26)	180.01 (6.38)	168.05 (5.41)	**<0.001**
BMI (kg·m^−2^)	23.66 (2.54)	24.06 (2.41)	21.65 (2.18)	24.04 (2.65)	24.32 (2.56)	21.63 (2.11)	**<0.001**
BF (%)	16.47 (5.17)	15.48 (4.52)	21.48 (5.33)	16.75 (5.07)	16.09 (4.68)	22.46 (4.66)	0.09
FFM (kg)	62.45 (9.27)	65.40 (6.44)	47.49 (6.55)	63.93 (8.17)	65.86 (6.02)	47.23 (4.40)	**<0.001**
HRmax (beats·min^−1^)	184.60 (9.79)	184.47 (9.88)	185.22 (9.33)	182.67 (10.27)	182.46 (10.30)	184.38 (9.97)	**<0.001**
RERmax	1.12 (0.04)	1.12 (0.04)	1.12 (0.04)	1.13 (0.04)	1.13 (0.05)	1.12 (0.03)	**<0.001**
VE/VCO_2_max	31.75 (3.89)	31.70 (3.83)	32.02 (4.22)	29.39 (4.18)	29.33 (4.17)	29.92 (4.28)	**<0.001**
f_R_ (breaths·min^−1^)	57.22 (9.11)	57.57 (9.22)	55.44 (8.31)	56.37 (9.32)	56.51 (9.47)	55.18 (7.92)	**0.004**
VO_2_max (mL·min^−1^·kg^−1^)	53.24 (7.12)	54.09 (6.92)	48.90 (6.54)	51.67 (7.86)	51.95 (7.96)	49.21 (6.53)	**<0.001**
O_2pulse_max (mL·beat^−1^)	21.58 (4.03)	22.69 (3.24)	15.95 (2.73)	21.66 (3.62)	22.28 (3.18)	16.31 (2.67)	0.53

Abbreviations: CPET, cardiopulmonary exercise test; BMI, body mass index; BF, body fat; FFM, fat-free mass; HRmax, maximal heart rate; RERmax, maximal respiratory exchange ratio; VE/VCO_2_max, maximal minute ventilation efficiency; f_R_, breathing frequency; VO_2_max, maximal oxygen uptake; O_2pulse_max, maximal oxygen pulse. Categorical data are presented as numbers (percentages). Continuous data are presented as mean (standard deviation). Differences between running and cycling CPET were calculated by Student *t*-test. Significant *p*-values (<0.05) are bolded.

**Table 2:** Comparison of performance of selected HRmax prediction models.

Reference	Equation			Performance in the Athletic Population					
		Predicted HRmax (Beats·min^−1^)		Difference from the Observed HRmax (Beats·min^−1^)		MAPE	RMSE (Beats·min^−1^)	%RMSE ‡	*p*-Value
		Mean (SD)	CI	Mean (SD)	CI				
Part A. Running CPET									
Nes et al. [14]	211 − 0.64 · age	188.23 (5.20)	188.07, 188.39	3.63 (8.98)	3.35, 3.91	4.31	9.69	5.25	**<0.001**
Machado et al. [29]	218 − 0.8 · age	189.54 (6.50)	189.34, 189.74	4.94 (9.24)	4.66, 5.23	4.69	10.47	5.67	**<0.001**
Tanaka et al. [2]	208 − 0.7 · age	183.09 (5.68)	182.92, 183.27	−1.50 (9.06)	−1.78, −1.22	3.96	9.18	4.97	**<0.001**
Fox et al. [15]	220 − age	184.42 (8.12)	184.17, 184.67	−0.18 (9.79)	−0.48, −0.12	4.25	9.79	5.13	0.38
Londeree et al. [30]	206.3 − 0.711 · age	181.00 (5.77)	180.82, 181.18	−3.59 (9.07)	−3.87, −3.31	4.17	9.76	5.29	**<0.001**
Inbar et al. [17]	205.8 − 0.685 · age	181.43 (5.56)	181.26, 161.60	−3.17 (9.04)	−3.45, −2.89	4.10	9.58	5.19	**<0.001**
Gellish et al. [31]	207 − 0.7 · age	182.09 (5.68)	181.92, 182.27	2.50 (9.06)	2.22, 2.78	4.03	9.40	5.09	**<0.001**
Arena et al. [16]	209.3 − 0.72 · age	183.68 (5.85)	183.50, 183.86	−0.91 (9.09)	−1.19, −0.63	3.95	9.13	4.95	**<0.001**
Part B. Cycling CPET									
Tanaka et al. [2]	208 − 0.7 · age	182.19 (6.32)	181.84, 182.54	−0.48 (9.24)	−0.99, −0.03	4.11	9.26	5.07	0.16
Fox et al. [15]	220 − age	183.12 (9.03)	182.62, 183.62	0.46 (10.06)	−0.09, 1.01	4.41	10.07	5.52	0.23
Londeree et al. [30]	206.3 − 0.711 · age	180.08 (6.42)	179.73, 180.43	−2.58 (9.26)	−3.09, −2.07	4.31	9.61	5.26	**<0.001**
Fairbarn et al. [18]	201 − 0.63 · age	177.77 (5.69)	177.46, 178.08	−4.90 (9.16)	−5.40, −4.40	4.68	10.38	5.68	**<0.001**
Arena et al. [16]	209.3 − 0.72 · age	182.75 (6.50)	182.39, 183.11	0.08 (9.28)	−0.43, 0.59	4.10	9.28	5.08	0.80

Abbreviations: HRmax, maximal heart rate; SD, standard deviation; CI, 95% confidence interval; MAPE, mean absolute percentage error; RMSE, root mean standard error; %RMSE, percentage of root mean square error; CPET, cardio-pulmonary exercise test. Running CPET, n = 4045; cycling CPET, n = 1268. Data are presented as mean (standard deviation) and 95% confidence intervals. Mean (standard deviation; 95% confidence interval) of observed HR_max_; running = 184.60 (9.79; 184.30, 184.90); cycling = 182.66 (10.28; 182.11, 183.24). Age is calculated in years. Differences between measured and predicted HR_max_ were calculated from the Student *t*-test. Significant *p*-values (<0.05) are bolded. Original derivation studies are presented in the Supplementary Materials File S3: Selected prediction models for maximal heart rate. Part A (upper) presents formulae for running and Part B (lower) for cycling. ‡ %RMSE = RMSE/median of observed.

## Data Availability

The data presented in this study are available on request from the corresponding author. The data are not publicly available due to not obtaining consent from respondents to publish the data.

## Supplementary Materials

The following supporting information can be downloaded at: https://www.mdpi.com/article/10.3390/jcm12082884/s1, Supplementary Materials File S1: TRIPOD Checklist: Prediction Model Development and Validation; Supplementary Materials File S2: Prediction performance of selected maximal heart rate prediction models; Supplementary Materials File S3: Selected prediction models for maximal heart rate. Reference [45] is cited in Supplementary Materials.
