# Supplementary material for: Validity of the Maximal Heart Rate Prediction Models among Runners and Cyclists
Source: J Clin Med. 2023 Apr 14;12(8):2884. doi: 10.3390/jcm12082884 (PMC10146295; doi:10.3390/jcm12082884)
Supplement: Supplementary file 1 [file jcm-12-02884-s001.zip › Supplementary Materials File S2.pdf]

Supplementary Material S2. Prediction performance of selected maximal heart rate prediction models.

Supplementary Material 2. Prediction performance of selected maximal heart rate prediction models. Abbreviations: HRmax, maximal heart rate. Data are presented in beats·min^{-1}. Dotted lines represent mean bias of agreement. Panels illustrate the performance of the following equations: A- Nes et al. (running CPET); B- Machado et al. (running CPET); C- Tanaka et al. (running CPET); D- Fox et al. (running CPET); E- Londeree et al. (running CPET); F- Inbar et al. (running CPET); G- Gellish et al. (running CPET); H- Arena et al. (running CPET); I- Tanaka et al. (cycling CPET); J- Fox et al. (cycling CPET); K- Londeree et al. (cycling CPET); L- Fairbarn et al. (cycling CPET); M- Arena et al. (cycling CPET).

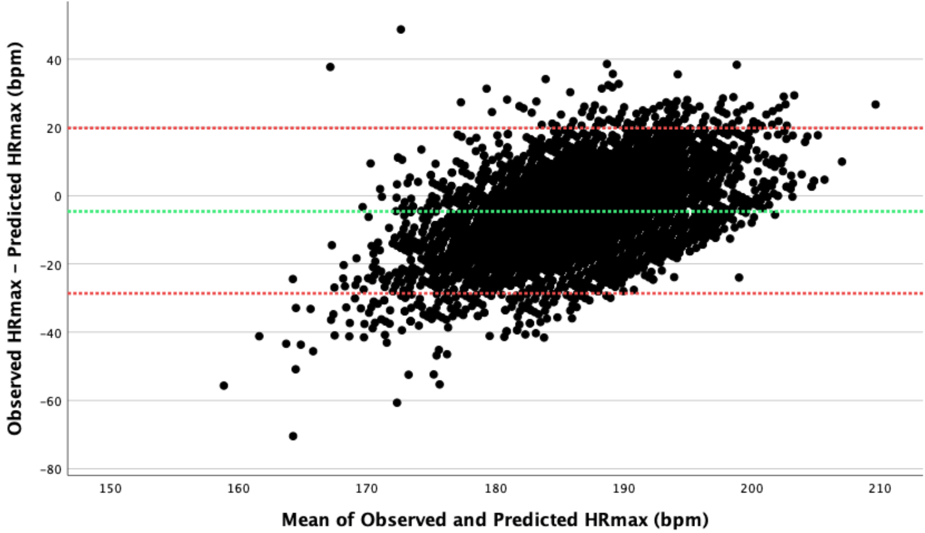

40
20
0
-20
-40
-60
-80
Observed HRmax – Predicted HRmax (bpm)
150
160
170
180
190
200
210
Mean of Observed and Predicted HRmax (bpm)

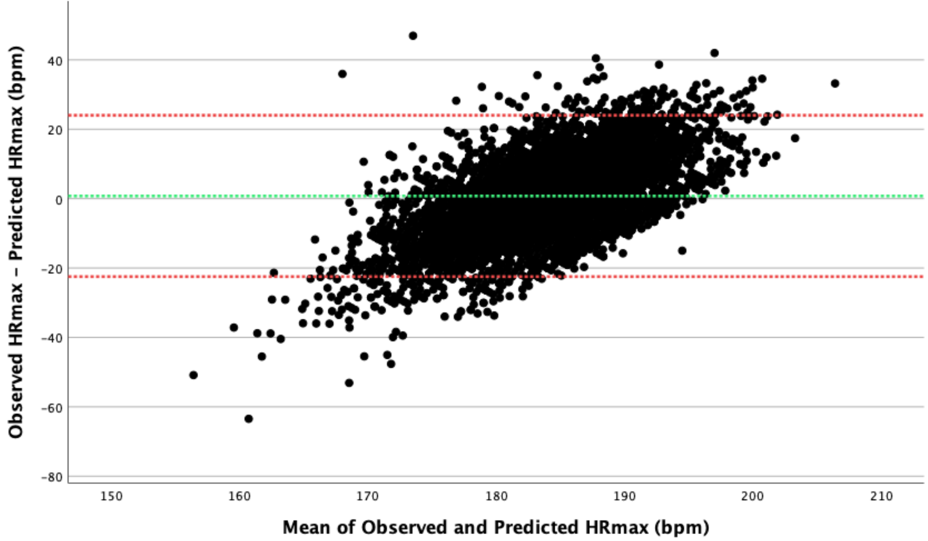

Observed HRmax – Predicted HRmax (bpm)
40
20
0
-20
-40
-60
-80
150
160
170
180
190
200
210
Mean of Observed and Predicted HRmax (bpm)

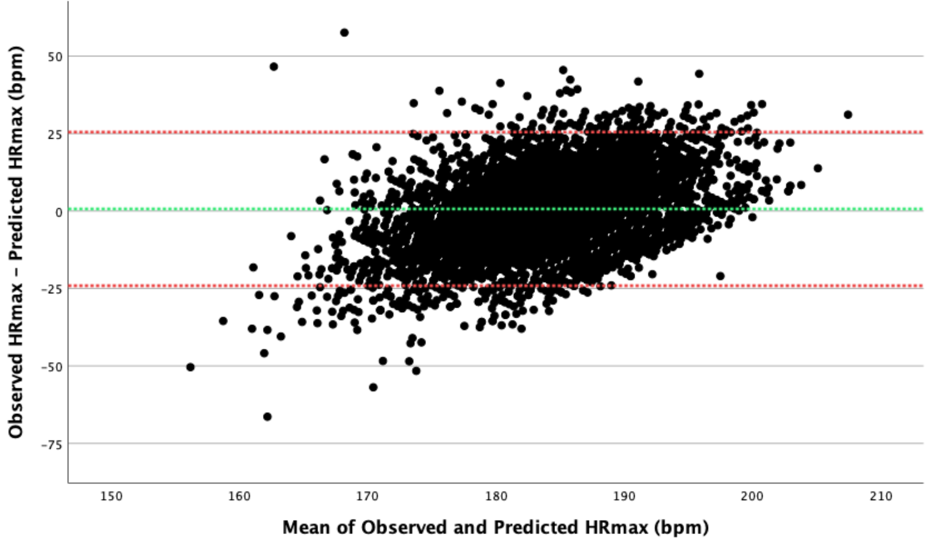

Observed HRmax – Predicted HRmax (bpm)
50
25
0
-25
-50
-75
150
160
170
180
190
200
210
Mean of Observed and Predicted HRmax (bpm)

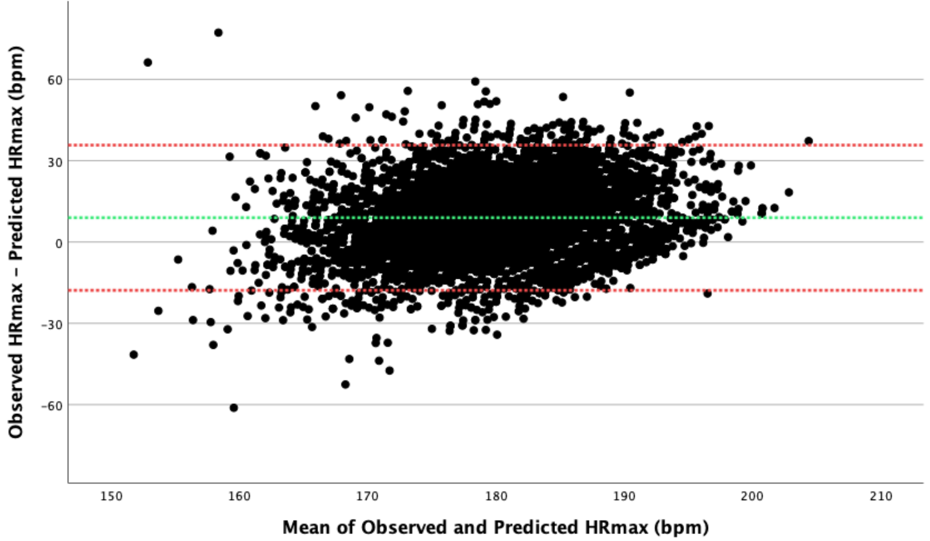

Observed HRmax – Predicted HRmax (bpm)
60
30
0
-30
-60
150
160
170
180
190
200
210
Mean of Observed and Predicted HRmax (bpm)

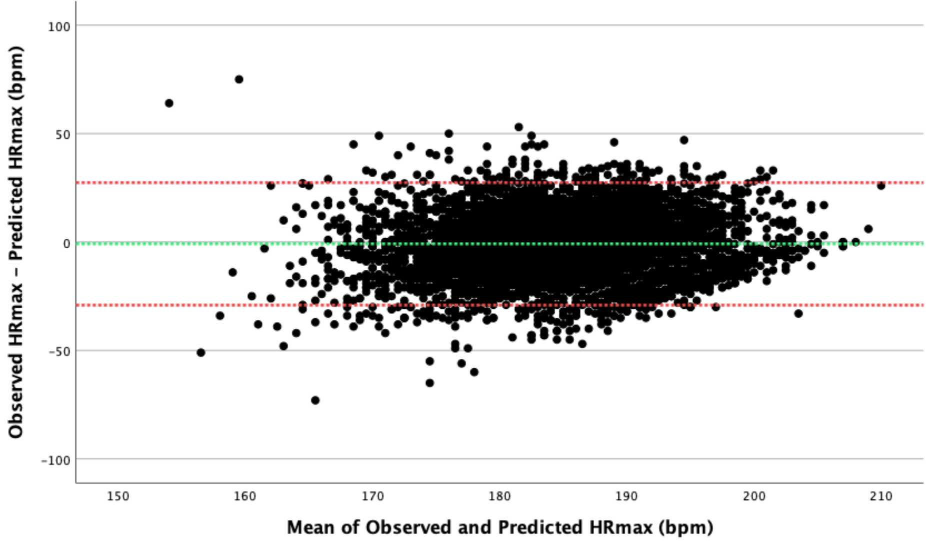

100
50
0
−50
−100
150
160
170
180
190
200
210
Observed HRmax − Predicted HRmax (bpm)
Mean of Observed and Predicted HRmax (bpm)

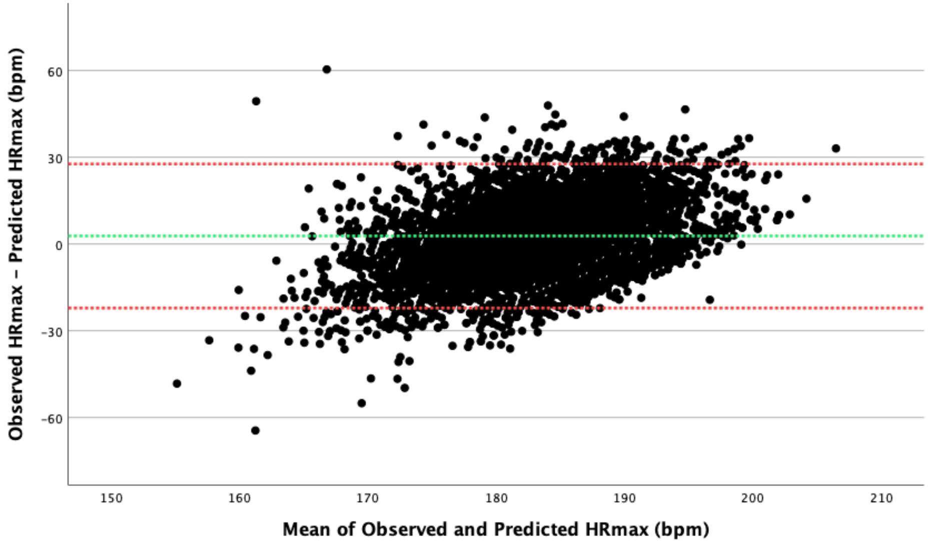

60
30
0
-30
-60
150
160
170
180
190
200
210
Observed HRmax – Predicted HRmax (bpm)
Mean of Observed and Predicted HRmax (bpm)

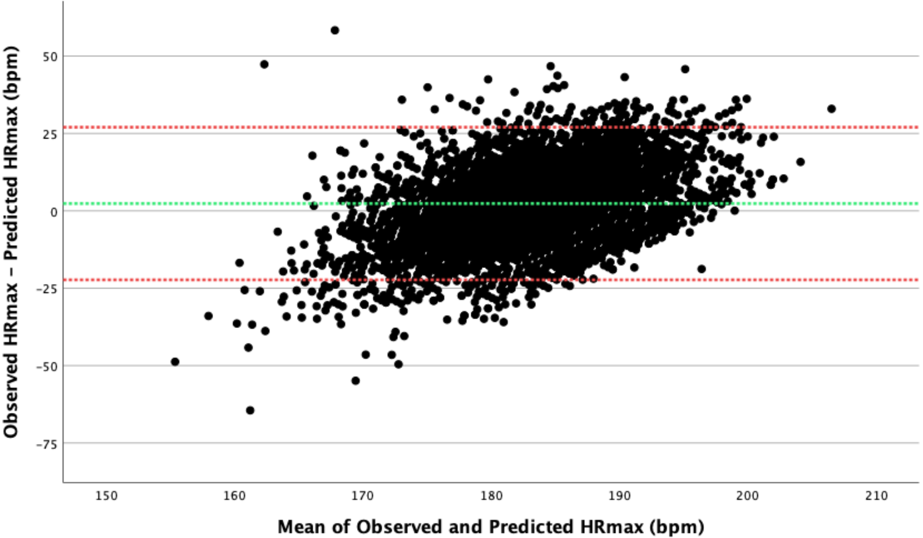
Observed HRmax – Predicted HRmax (bpm)
50
25
0
-25
-50
-75
150
160
170
180
190
200
210
Mean of Observed and Predicted HRmax (bpm)

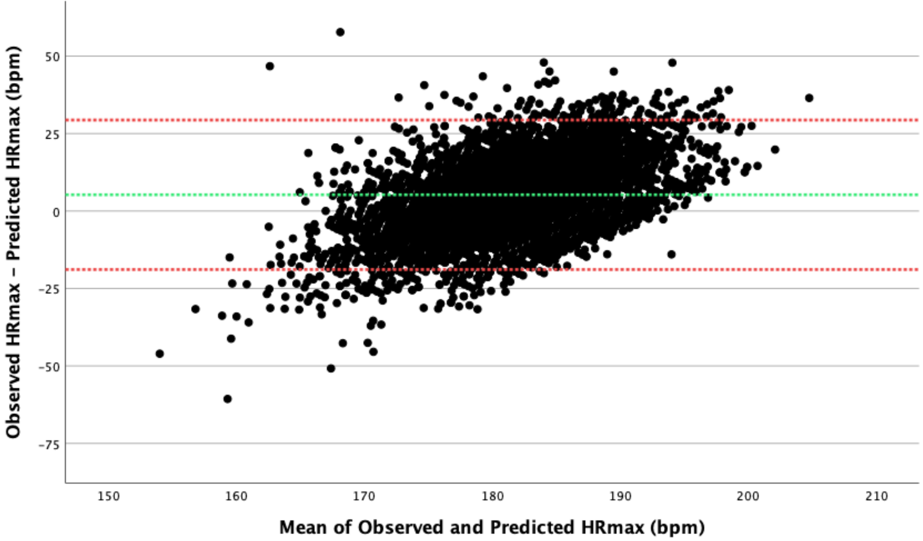

Observed HRmax − Predicted HRmax (bpm)
50
25
0
−25
−50
−75
150
160
170
180
190
200
210
Mean of Observed and Predicted HRmax (bpm)

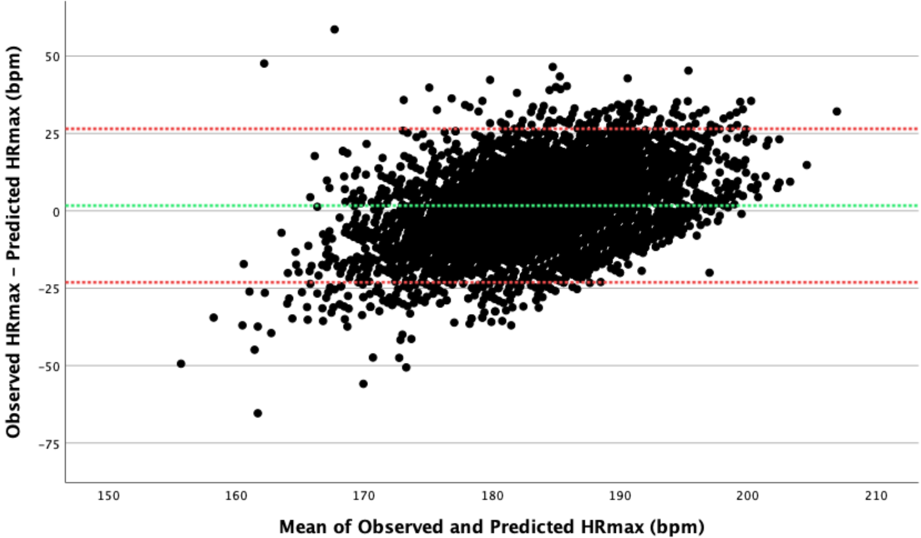

Observed HRmax − Predicted HRmax (bpm)
50
25
0
-25
-50
-75
150
160
170
180
190
200
210
Mean of Observed and Predicted HRmax (bpm)

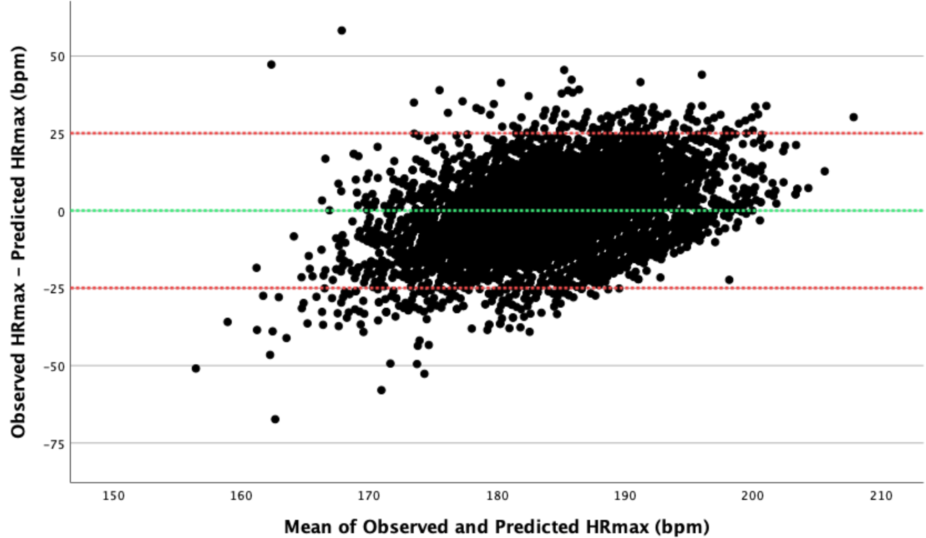

Observed HRmax – Predicted HRmax (bpm)
50
25
0
-25
-50
-75
150
160
170
180
190
200
210
Mean of Observed and Predicted HRmax (bpm)

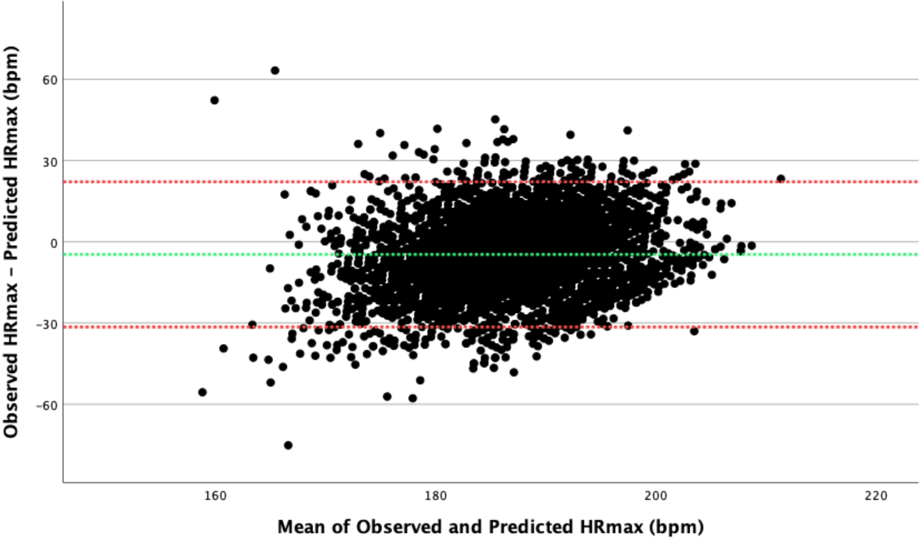

60
30
0
-30
-60
160
180
200
220
Observed HRmax – Predicted HRmax (bpm)
Mean of Observed and Predicted HRmax (bpm)

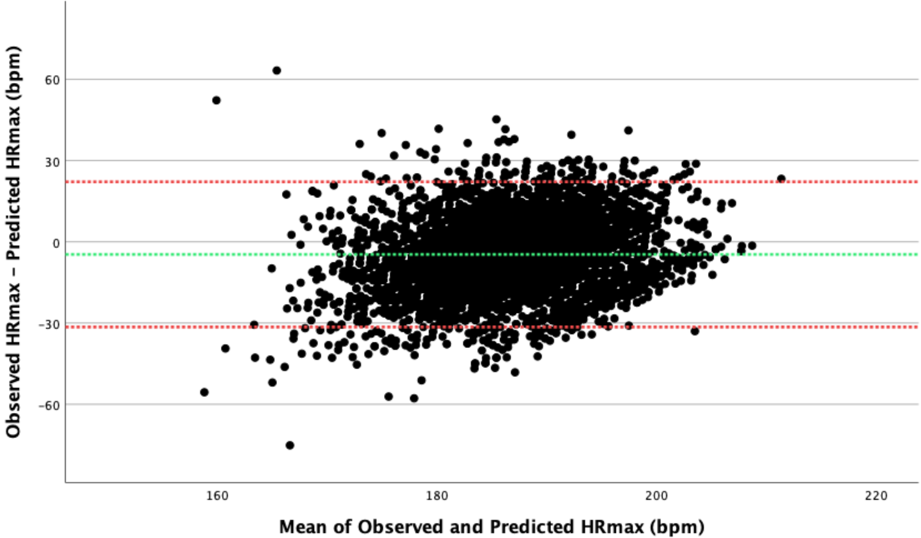

Observed HRmax – Predicted HRmax (bpm)
60
30
0
-30
-60
160
180
200
220
Mean of Observed and Predicted HRmax (bpm)

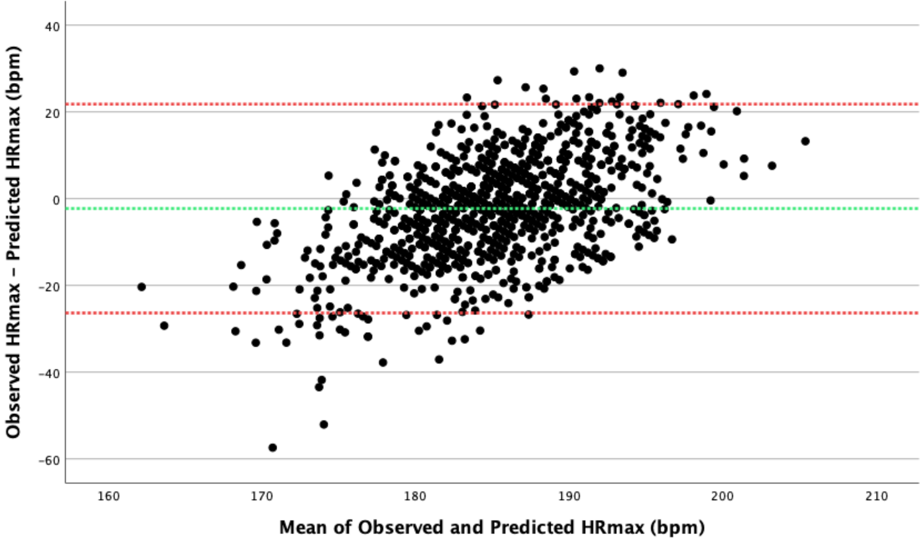

40
20
0
-20
-40
-60
160
170
180
190
200
210
Observed HRmax – Predicted HRmax (bpm)
Mean of Observed and Predicted HRmax (bpm)

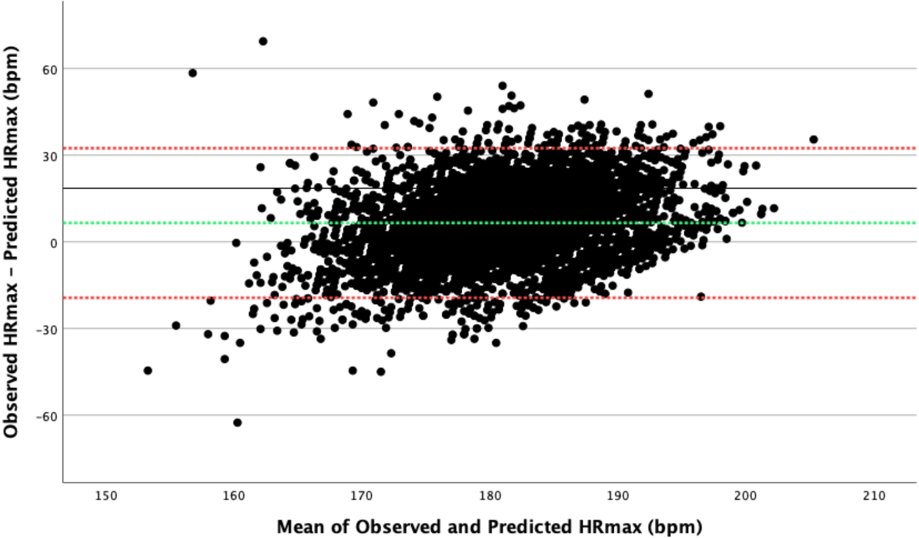
Observed HRmax – Predicted HRmax (bpm)
60
30
0
-30
-60
150
160
170
180
190
200
210
Mean of Observed and Predicted HRmax (bpm)

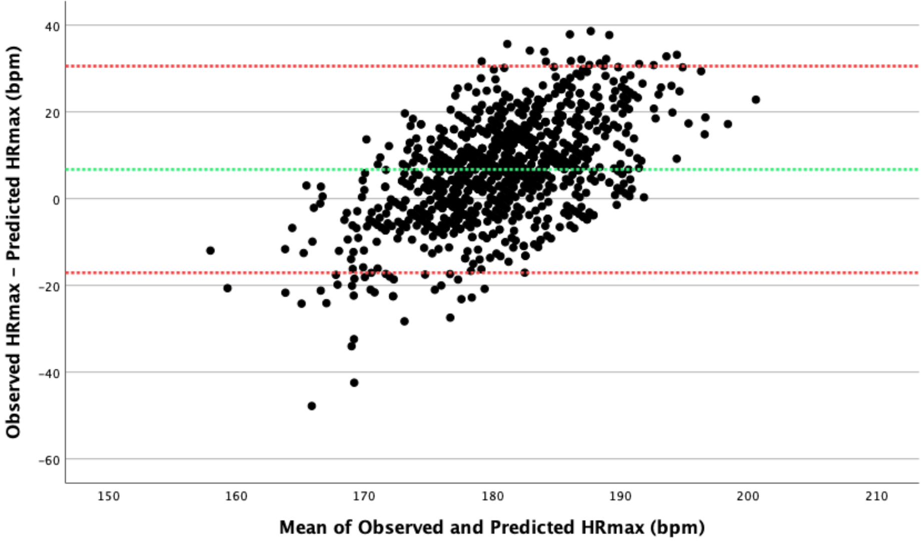
40
20
0
-20
-40
-60
Observed HRmax – Predicted HRmax (bpm)
150
160
170
180
190
200
210
Mean of Observed and Predicted HRmax (bpm)

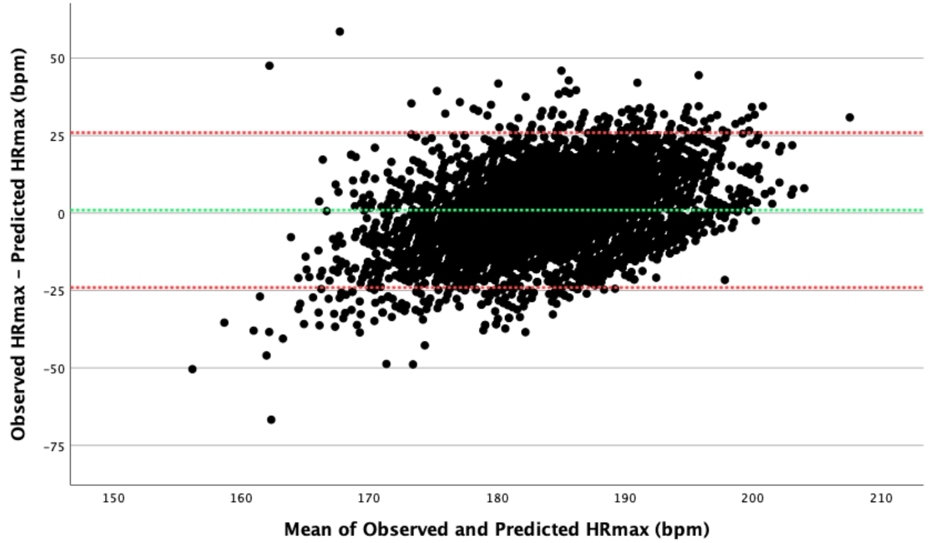
Observed HRmax – Predicted HRmax (bpm)
50
25
0
-25
-50
-75
150
160
170
180
190
200
210
Mean of Observed and Predicted HRmax (bpm)

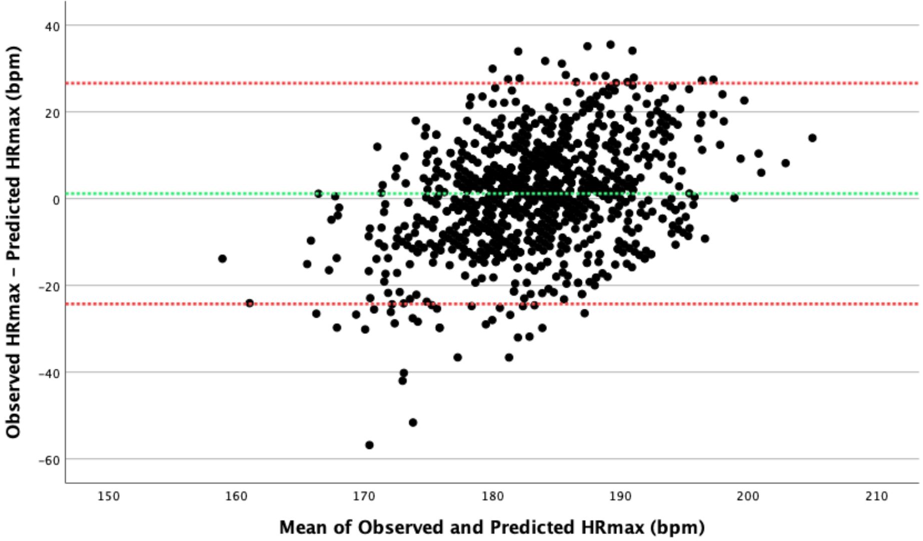

40
20
0
-20
-40
-60
150
160
170
180
190
200
210
Observed HRmax – Predicted HRmax (bpm)
Mean of Observed and Predicted HRmax (bpm)

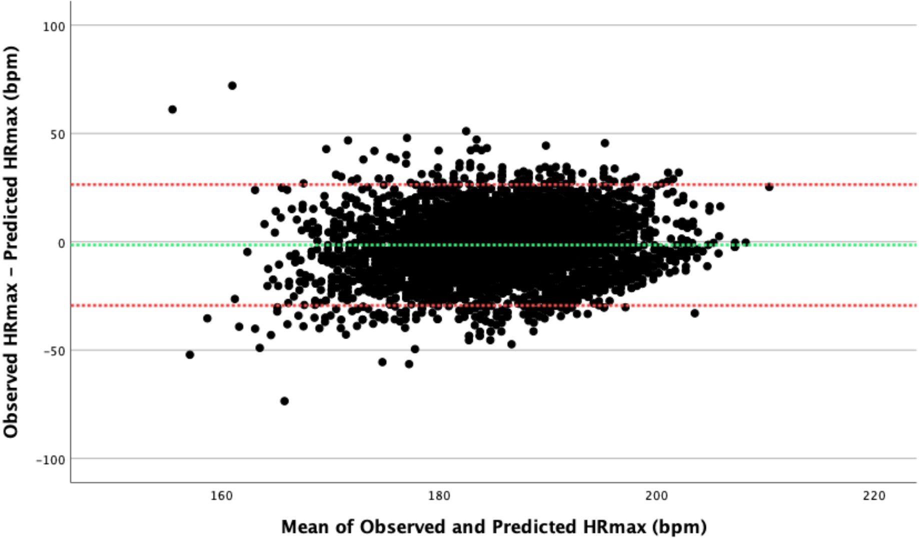

100
50
0
-50
-100
Observed HRmax – Predicted HRmax (bpm)
160
180
200
220
Mean of Observed and Predicted HRmax (bpm)

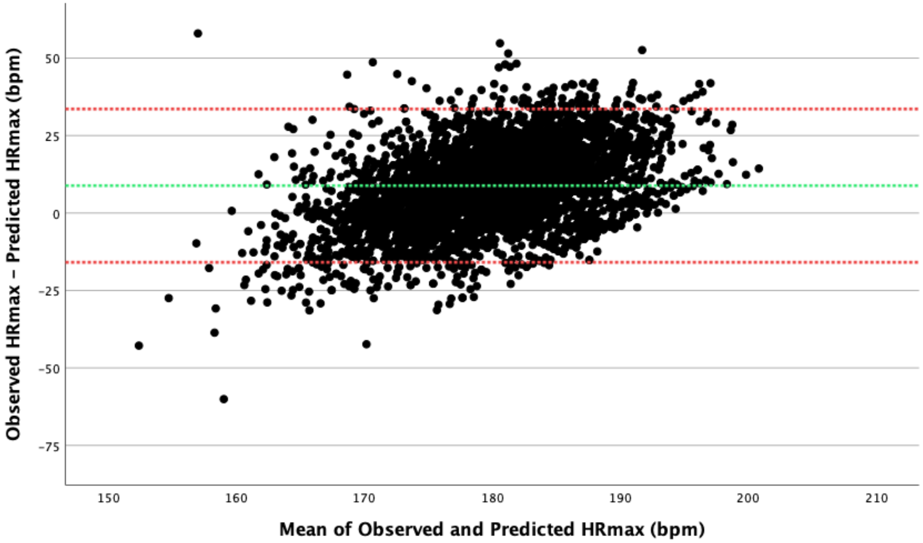

Observed HRmax – Predicted HRmax (bpm)
50
25
0
-25
-50
-75
150
160
170
180
190
200
210
Mean of Observed and Predicted HRmax (bpm)

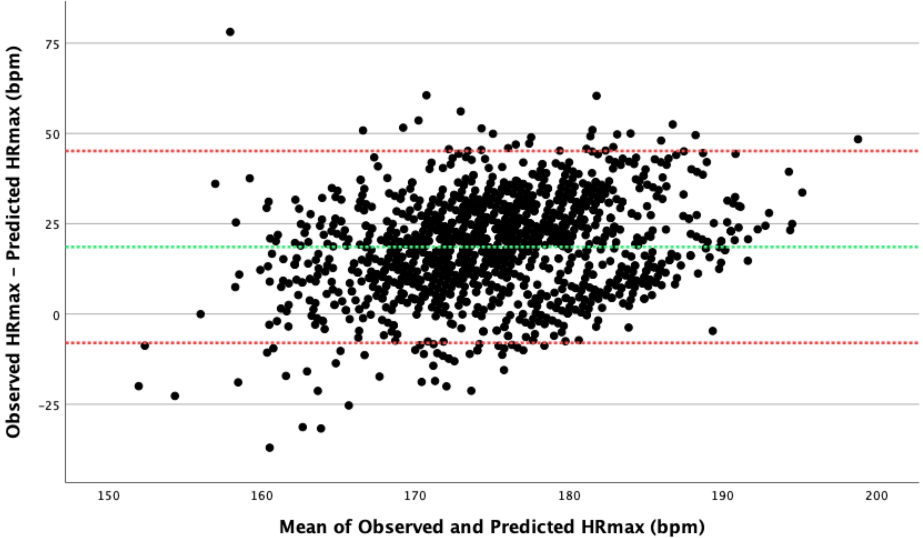

Observed HRmax – Predicted HRmax (bpm)
75
50
25
0
–25
150
160
170
180
190
200
Mean of Observed and Predicted HRmax (bpm)
