# Supplementary material for: Validity of the Maximal Heart Rate Prediction Models among Runners and Cyclists
Source: J Clin Med. 2023 Apr 14;12(8):2884. doi: 10.3390/jcm12082884 (PMC10146295; doi:10.3390/jcm12082884)
Supplement: Supplementary file 1 [file jcm-12-02884-s001.zip › Supplementary Materials File S3.pdf]

Supplementary Material S3. Selected prediction models for maximal heart rate.

References to the original studies with prediction models selected for our external validation. All references are cited in the same order as they are listed in Table 2 in the main body of the manuscript:

1. Nes BM, Janszky I, Wisloff U, Stoylen A, Karlsen T. Age-predicted maximal heart rate in healthy subjects: The HUNT fitness study. Scand J Med Sci Sports. 2013;23(6):697-704.
2. Machado FA, Kravchychyn ACP, Peserico CS, da Silva DF, Mezzaroba PV. A new age-based equation for predicting maximum heart rate in endurance-trained runners. Revista Brasileira de Ciências do Esporte. 2018;40(1):100-5.
3. Tanaka H, Monahan KD, Seals DR. Age-predicted maximal heart rate revisited. J Am Coll Cardiol. 2001;37(1):153-6.
4. Fox S. Physical activity and the prevention of coronary heart disease. Annals of clinical research. 1968;3 6:404-32.
5. Londeree BR, Moeschberger ML. Effect of Age and Other Factors on Maximal Heart Rate. Research Quarterly for Exercise and Sport. 1982;53(4):297-304.
6. Inbar O, Oren AMI, Scheinowitz M, Rotstein A, Dlin R, Casaburi R. Normal cardiopulmonary responses during incremental exercise in 20??? to 70-yr-old men. Medicine & Science in sports & Exercise. 1994;26(5).
7. Gellish RL, Goslin BR, Olson RE, McDonald A, Russi GD, Moudgil VK. Longitudinal modeling of the relationship between age and maximal heart rate. Med Sci Sports Exerc. 2007;39(5):822-9.
8. Arena R, Myers J, Kaminsky LA. Revisiting age-predicted maximal heart rate: Can it be used as a valid measure of effort? Am Heart J. 2016;173:49-56.
9. Itoh H, Ajisaka R, Koike A, Makita S, Omiya K, Kato Y, et al. Heart rate and blood pressure response to ramp exercise and exercise capacity in relation to age, gender, and mode of exercise in a healthy population. J Cardiol. 2013;61(1):71-8.
10. Fairbarn MS, Blackie SP, McElvaney NG, Wiggs BR, Pare PD, Pardy RL. Prediction of heart rate and oxygen uptake during incremental and maximal exercise in healthy adults. Chest. 1994;105(5):1365-9.
